# Supplementary material for: Phytochemistry, Pharmacology and Mode of Action of the Anti-Bacterial Artemisia Plants
Source: Bioengineering (Basel). 2023 May 23;10(6):633. doi: 10.3390/bioengineering10060633 (PMC10295440; doi:10.3390/bioengineering10060633)
Supplement: Supplementary file 1 [file bioengineering-10-00633-s001.zip › bioengineering-2402571-supplementary.pdf]

Phytochemistry, pharmacology and mode of action of the anti-bacterial Artemisia plants

Khotibul Umam[1,2,3,4], Ching-Shan Feng[1], Greta Yang[1], Ping-Chen Tu[5], Chih-Yu Lin[1], Meng-Ting Yang[1], Tien-Fen Kuo[1], Wen-Chin Yang[1,2,3,6,7*], and Hieu Tran Nguyen Minh[1*]

Supplementary Materials

Table S1. Degree of anti-bacterial activity of different compounds based on the MIC value against pathogens (modified from [61]).

**Activity**	**MIC (μg/ml)**
Very high activity	≤ 40
High activity	< 40 to ≤ 80
Moderate activity	< 80 to ≤ 160
Low activity	< 160 to ≤ 300
Very low activity	> 300

Table S2. Ranking of the anti-bacterial activity of the 75 compounds based on the MIC value against pathogens.

**Ranking**	**Compounds**
Very high	phytol (**46**), α-amyrin (**47**), betulinic acid (**48**), acacetin (**50**), 12α,4α-dihydroxybishopsolicepolide (**39**), scopoletin (**61**), chrysoplenol B (**54**), jaceosidin (**59**), jaceidine (**60**), vulgarone B sesquiterpenoid (**34**), (*Z*)-2-(Hexa-2,4-diyn-1-ylidene)-1,6-dioxaspiro(4.5)dec-3-ene (**68**), (*E*)-2-(Hexa-2,4-diyn-1-ylidene)-1,6-dioxaspiro(4.5)dec-3-ene (**69**), dehydroleucodine (**38**)
High-Very high	nerol (**20**)
Moderate-Very high	artemisia ketone (**18**), sabinene (**1**), germacrene B (**16**), p-cymene (**5**), germacrene D (**17**)
Low-Very high	α-elemene (**11**), α-pinene (**9**), β-pinene (**10**), α-farnesene (**13**), carveol (**26**)
Very low-Very high	α-farnesene (**13**), 1,8-cineole/ eucalyptol (**28**), methyl linolenate (**72**), diisooctyl phthalate (**66**), camphor (**33**),

	ursolic acid (**49**), β-caryophyllene (**12**), *α*-terpineol (**24**), (+)-borneol (**31**), limonene (**6** & **7**)
High	thymol (**25**)
Moderate-high	integracid (**67**), capillin (**64**)
Low-High	camphene (**8**), zerumbone sesquiterpenoid (**37**)
Very low-High	(*Z*)-chrysanthenyl acetate (**27**), terpinen-4-ol (**23**)
Moderate	piperitone (**22**), artemisinin (**44**), estragole (**73**), (+)-*(S)*-dihydro-*ar*-turmerone (**36**)
Low-Moderate	ponticaepoxide (**70**), *α*-curcumene (**14**), (+)-*(S)-ar*-turmerone sesquiterpenoid (**35**), grandisol (**21**)
Very low-Moderate	myrcene (**2**), *α*-thujone (**29**), β-thujone (**30**), dihydro-*ar*-curcumene (**15**), (*E*)-β-ocimene (**3**), (*Z*)-β-ocimene (**4**)
Very low-low	eugenol (**75**), (-) borneol (**32**)
Low	chrysosplenol D (**55**), linalool (**19**)
Very low	artesunate (**45**), (+)-*threo*-(5*E*)-trideca-1,5- dien-7,9,11-triyne-3,4-diol (**71**), casticin (**51**), 2,4-dihydroxy-6-methoxyacetophenone (**63**), rosmarinic acid (**74**)
No ranking (no assessment of MICs)	chrysosplenetin (**52**), chrysoeriol (**53**), penduletin (**56**), artemetin (**57**), pachypodol (**58**); 1,3,8-trihydroxyeudesm-4-en-7*α*,11β*H*-12,6*α*-olide (**40**); 3*α*,8β-dihydroxygermacr-4(15),9(10)-dien-7β,11*αH*,12,6*α*-olide (**41**); 1β,8*α*-dihydroxy-11*α*,13-dihydrobalchanin (**42**); 11-epiartapshin (**43**); 5-β-D-glucopyranosyloxy-7-methoxy-6*H*-benzopyran-2-one (**62**); benzoic acid, *p*-(β-D-glucopyranosyloxy)-methyl ester (**65**)
